# Challenges in prenatal and pre-implantation genetic diagnosis studies

**DOI:** 10.1186/1755-8166-7-S1-I50

**Published:** 2014-01-21

**Authors:** Prochi Madon

**Affiliations:** 1 Department of Assisted Reproduction and Genetics, Jaslok Hospital and Research Centre, Mumbai, India

## 

Prenatal diagnosis (PND) of chromosomal anomalies by karyotyping and fluorescence *in situ* hybridization (FISH) is a routine procedure in many cytogenetic laboratories. However, PND of single gene disorders is available in only a few centres specializing in molecular genetic testing in India. Preimplantation genetic diagnosis (PGD) is a state-of the art technique involving biopsy of a single cell from a cleavage stage embryo obtained after intra-cytoplasmic sperm injection (ICSI). This is an additional step during in-vitro fertilization (IVF). The biopsied cell is then fixed on a slide in a critical step where the cytoplasm is removed to expose the nucleus. Single cells from many embryos of the couple are fixed and then screened for common aneuploidies by FISH using multicolour DNA probes. A maximum of 5 chromosomes can be checked in 1 hybridization. Hence the probes are stripped and slides are rehybridized in 2-4 rounds to study more chromosomes within a limited time frame. Only the chromosomally normal embryos are transferred or cryopreserved for a subsequent transfer.

Karyotyping of couples with recurrent early miscarriages helps to identify the cause if the husband or wife is a carrier of a balanced translocation. PGD can help such couples to have a healthy baby earlier than by normal chance. Specific FISH probes have to be ordered especially for reciprocal translocations and a pre-PGD work up is important. This helped to identify an additional cryptic translocation between chromosomes 9 and 12 in a case from a neighbouring country, where the husband had inversion 12. Hence PGD included testing of chromosome 9 in this case. The wife has an ongoing pregnancy in the first attempt. Currently, ours is the only centre in India which offers PGD even for reciprocal/ Robertsonian translocations and microdeletions. We have had success with the birth of healthy babies.

PGD for single gene disorders is yet in its infancy in our country. It involves genetic testing from DNA extracted from a single cell. Pre-PGD work up is more demanding, especially in cases where a HLA matched normal savior sibling is desired to cure a child affected with a hematological disorder such as Thalassemia.

